# Acid challenge exacerbates activation of matrix metalloproteinases in permanent teeth undergoing radiotherapy

**DOI:** 10.1590/1807-3107bor-2024.vol38.0034

**Published:** 2024-05-13

**Authors:** Alexandra Mussolino de QUEIROZ, Claudia María Carpio BONILLA, Taíssa Cássia de Souza FURTADO, Regina Guenka PALMA-DIBB, Harley Francisco de OLIVEIRA, Maya Fernanda Manfrin ARNEZ, Fabrício Kitazono de CARVALHO, Francisco Wanderley Garcia PAULA-SILVA

**Affiliations:** (a) Universidade de São Paulo – USP, School of Dentistry of Ribeirão Preto, Department of Pediatric Clinics, Ribeirão Preto, SP, Brazil.; (b) Universidade de São Paulo – USP, School of Dentistry of Ribeirão Preto, Department of Restorative Dentistry, Ribeirão Preto, SP, Brazil.

**Keywords:** Radiotherapy, Matrix Metalloproteinases, Tooth Demineralization

## Abstract

The aim of this study was to investigate the effect of acid challenge on the activation of matrix metalloproteinases (MMPs) in the Dentinoenamel junction of primary and permanent teeth submitted to radiotherapy. For this purpose, a total of 178 dental fragments obtained from molars were used, and randomly divided into 2 groups (primary and permanent teeth) / 4 experimental subgroups (irradiated and non-irradiated, demineralized and non-demineralized). The fragments were exposed to radiation, with a dose fraction of 2 Gy, for 5 consecutive days, until a total dose of 60 Gy was reached, with a total of 30 cycles, for 6 weeks. To determine the activity of MMPs on the dentinoenamel junction (DEJ), in situ zymography assays on 0.6mm dental fragments were performed. To assess whether MMP activity would be impacted by an acidic environment, the fragments were placed in a demineralizing solution (pH of 4.8). The finding was that irradiation activated MMPs in DEJ and these effects were more evident in permanent when compared with primary teeth. When the effect of an acid challenge on MMPs activity was investigated, demineralization was observed not to increase MMPs activity in non-irradiated teeth, but it did increase MMPs activity in irradiated teeth. In conclusion, an acid challenge was found to exacerbate activation of MMPs in DEJ of permanent teeth submitted to irradiation, but not in primary teeth.

## Introduction

Cancer in the head and neck region has a prevalence of 29.2 people per 100,000 according to the US National Cancer Institute.^1^ Salivary gland tumors comprise approximately 3% to 10% of all head and neck neoplasms, and are more uncommon in children than in adults. The choice of treatment for cancer in this region, based on histology and differentiation, may be surgery, irradiation, chemotherapy or a combination of these treatment methods.^1,2^ Radiotherapy is a challenging treatment that causes serious secondary consequences such as destruction of teeth and oral function, consequently influencing the quality of life of people.^1^

Patients undergoing radiotherapy in the head and neck region present dental decay that could result in enamel delamination from dentin due to instability at the dentinoenamel junction (DEJ).^3-5^ Enamel delamination is explained by the action of radiation capable of causing lytic changes in collagen polypeptides and activation of matrix metalloproteinases (MMPs). These are proteases responsible for degradation of the extracellular matrix in teeth exposed to radiation, thus resulting in degradation of the organic components of enamel and DEJ.^6,7^ MMPs play important roles in different processes associated with teeth, including the progression of caries lesions and dental erosion.^8^ MMPs are secreted as inactive precursors and require activation to enable them to degrade extracellular matrix components. Purified human and salivary MMPs (-2, -8, -9) are activated at low pH (4.5).^9^

Apart from the direct effects on dental structures, head and neck radiotherapy also causes dry mouth, reduced salivary flow, change in salivary composition, changes in oral microbiota, trismus, reduced remineralization and consequently increased susceptibility to tooth decay and erosion.^10-12^ Changes in oral hygiene, consumption of a soft, carbohydrate-rich diet, consumption of acidic products to alleviate dry mouth symptoms may also favor the increase in these lesions.^1,10^ Moreover, changes in the properties of enamel and dentin caused by irradiation are pointed out; these can lead to biomechanical failure, and thus, loss of enamel and progressive tooth decay or radiation decay,^5^ characterized by enamel erosion and dentin exposure that occur mainly in the cervical areas, and on occlusal and incisal edges.^13^

During the carious process of progressing to carious injury, the acidic environment created by bacterial acids can lead to activation of MMPs. Although activated MMPs are stable at acidic pH, they work best at neutral pH. During enamel demineralization, hydroxyapatite is solubilized by organic acids produced by bacteria; these can diffuse into calcified tissues when the local pH drops to below 5.5, leading to mineral tissue dissolution. In dentin, cariogenic bacteria do not degrade the dentin matrix after demineralization. Demineralization is characterized by destruction of the organic matrix of dentin, caused by bacterial proteases, mediated by MMP. MMPs present in dentin are produced by odontoblasts and are involved in the process of dentin formation.^14^Low pH, therefore, causes dentin demineralization, exposure of collagen fibrils, and, concomitantly, dentin and/or salivary MMPs are activated. At low pH, MMPs have little activity, however, as the pH increases, activity of the MMPs increases, breaking down the collagen matrix exposed by demineralization, and allowing progression and the loss of dentin.^9^

As acid challenges and carious lesions occur commonly in patients undergoing radiotherapy, it is hypothesized that tooth decay might involve the activation of MMPs in teeth submitted to radiation. Thus, the aim of this study was to investigate the effect of acid challenge on the activation of MMPs in the DEJ of primary and permanent teeth of patients submitted to radiotherapy. The null hypothesis tested was that acid challenge would not impact MMP activation in irradiated deciduous and permanent teeth.

## Methodology

The present research project was approved by the Ethics Committee of the School of Dentistry of Ribeirão Preto at University of São Paulo (#43861415.0.0000.5419). The sample consisted of 12 mandibular permanent and 12 mandibular primary human molars. Initially, the teeth were sectioned 1 mm below the enamel-cementum junction, to remove the remaining root. Then, the teeth were sectioned in the mesial/distal direction, resulting in two halves, one corresponding to the labial and the other to lingual portion, to produce a total of 48 hemisections. Twenty four hemisections among them were irradiated and 24 remained non-irradiated. The irradiated hemisections were treated to simulate characteristics of radiotherapy for head and neck cancer, as follows: a cumulative dose of 2 Gy fractions was applied for 5 consecutive days until a total dose of 60 Gy was reached, i.e., a total of 30 cycles of irradiation over 6 weeks.^15,16^ The specimens were placed in a 24-well acrylic cell culture plate, and each well was filled with artificial saliva in a way that all specimens could receive the same direct ionizing radiation per unit area.

Subsequently, the irradiated and non-irradiated hemisections were cut along the tooth axis to produce slices of 0.6 mm (n = 96 slices) as previously described.^6,7^ In order to assess whether the activity of MMPs would be changed when submitted to an acid challenge, the fragments were placed in Marquezan demineralizing solution (acetic acid - 50mM, CaCl_2_ - 2.2mM, and NaH_2_PO_4_ - 2.2 mM adjusted to a 4.8 pH with NaOH).^17^ Twenty µl of the solution was applied to the fragments for one minute, and later, the fragment was washed with deionized water at room temperature for one minute. The findings of pilot studies have shown that one minute was not long enough to jeopardize the mineral content of the DEJ, however, it enabled activation of enzymes within the matrix. A long term exposure resulted in loss of mineral content, huge disorganization of the matrix and made it impossible to evaluate gelatinolytic activity (data not shown).

### In situ Zymography

The gelatinolytic activity was determined by in situ zymography for primary and permanent teeth that either received or did not receive exposure to acid and radiotherapy (n = 6 slices per group). The tooth sections were bonded to glass slides with cyanoacrylate and then were immersed in sodium borohydride (1 mg/ml; Sigma) for 15 minutes (3x), washed in phosphate buffered saline (PBS), and incubated in a medium containing a gelatinous substrate bound to fluorescein isothiocyanate (DQ ™ Gelatin, Molecular Probes, Eugene, USA), dissolved in PBS at a concentration of 1 mg/ml, at 37°C in a humidified dark chamber, for 3 hours. Tooth slices 0.6 mm thick allowed the incubation period to be significantly reduced due to the high amount of enzyme in the slices, and to reduce the amount of background noise to enhance the signal detected in fluorescence microscopy due to gelatin degradation.^18,19^ Additional slices (n = 6 slices per group) were pre-incubated in 20 mM ethylenediaminetetraacetic acid (EDTA, Sigma) for 1 hour and then incubated in the gelatinous substrate to verify whether the enzymatic activity was caused by MMPs.

**Figure 1:**
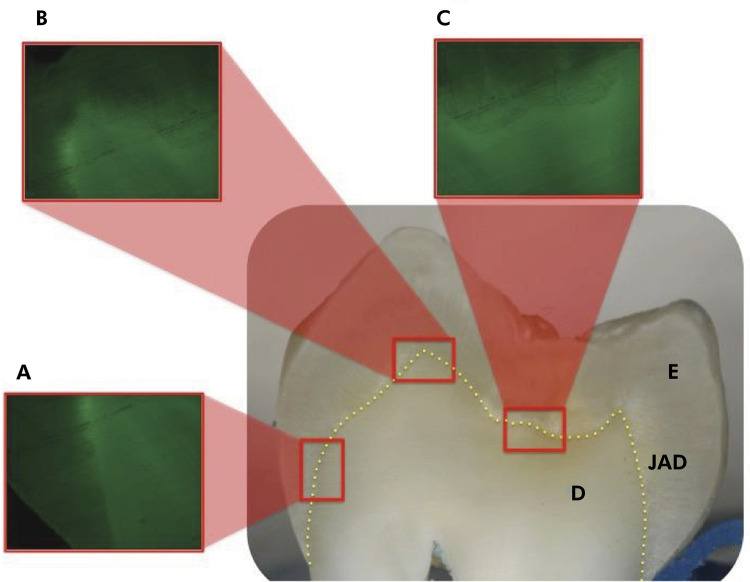
. Image of the regions evaluated in the study. (A) cervical, (B) cusp and (C) groove; enamel and dentin contiguous to enamel dentin junction. E, enamel; D, dentin; dentinoenamel junction (DEJ).

Gelatinolytic activity was evaluated in three regions close to the DEJ (cervical, cusp and groove) using a rectangle tool to draw a standardized area (Figure 1). Firstly, the regions were photographed under a fluorescence microscope at 1.25x and 5x magnifications using the Alexa Fluor 43HE filter (FT 570, BP 550/25, BP 605/70, Carl Zeiss, Germany). Subsequently, the 5x magnification images were analyzed by densitometry using the ImageJ software (National Institutes of Health, Bethesda, MD, USA). For quantification of gelatinolytic activity in the tooth sections, a representative standardized area was initially established, in which the fluorescent spots were quantified and expressed as arbitrary fluorescence units per mm^2^. One analysis in each region (cervical, cusp and groove) was performed in each specimen.

Data distribution was analyzed by the D’Agostino-Pearson test. Since data were not normally distributed, the groups were compared using the nonparametric Friedman test, followed by Dunn’s posttest (α = 0.05). All analyses were performed using GraphPad software Prism 9.4.1 (GraphPad Software, Inc., San Diego, USA).

## Results

**Figure 2:**
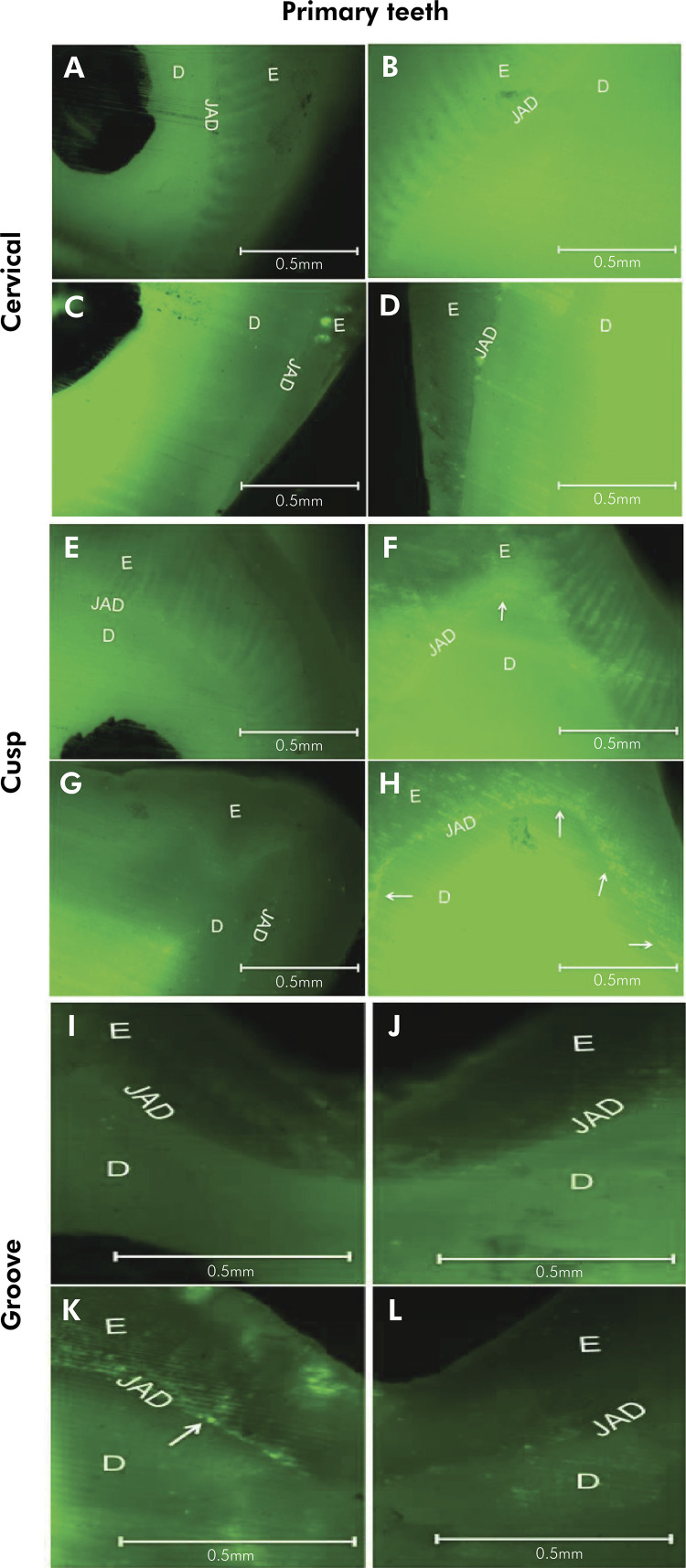
. Fluorescence microscopy revealed gelatinolytic activity in the regions of enamel (E), dentin (D) and dentinoenamel junction (DEJ), in human primary teeth. (A) Cervical region (5x), showing low activity of MMPs, (B) Cervical region (5x) previously demineralized at a pH of 4.8, showing low activity of MMPs, (C) Cervical region of a sectioned crown irradiated (5x) showing the activity of the MMPs, (D) Cervical region of an irradiated sectioned crown (5x), previously demineralized at a pH of 4.8 showing an activity of the MMPs, (E) Cusp (5x) showing the low activity of MMPs, (F) Cusp (5x) previously demineralized at a pH of 4.8, showing low activity of MMPs, (G) Cusp of an irradiated sectioned crown (5x) showing MMP activity, (H) Cusp of an irradiated sectioned crown (5x) previously demineralized at a pH of 4.8, showing MMP activity, (I) Groove region (5x) showing low MMP activity, (J) Groove region ( 5x), previously demineralized at a pH of 4.8, showing low activity of MMPs, (K) Groove region of an irradiated sectioned crown (5x), previously demineralized at a pH of 4.8, showing an activity of MMPs, (L) Groove region of an irradiated sectioned crown (5x), showing the activity of MMPs. Bar = 0.5 mm (A, B, C, D, E, F, G, H, I, J, K and L).

Acid challenge did not alter the activity of MMPs in non-irradiated primary teeth when compared with healthy teeth (that did not receive irradiation or acid challenge), in the cervical, cusp and groove regions. Likewise, for irradiated primary teeth, there was no change in integrated density of fluorescent signal/mm^2^, when compared with teeth either submitted to, or not submitted to acid challenge (Figure 2 and Table 1).

**Table 1:** Comparison of gelatinolytic activity of MMPs in irradiated and / or demineralized primary teeth, performed by means of in situ zymography. The integrated density of fluorescent signal/mm^2^ (median and interquartile range) in the cervical region, at the cusp and at the groove was measured at the same depth and in the same area.

Variable	Cervical	Cusp	Groove

	arbitrary units (mm^2^)	arbitrary units (mm^2^)	arbitrary units (mm^2^)
Healthy	24.485 (17.546–31.096)	26.765 (18.570–31.324)	24.738 (17.885–36.829)
Demineralized	28.440 (18.771–35.160)	19.444 (14.028–32.835)	20.895 (14.763–27.323)
Irradiated	26.076 (18.724–31.123)	17.159 (13.964–52.665)	20.377 (17.398–65.167)
Irradiated + Demineralized	24.941 (17.913–63.324)	35.599 (19.415–58.334)	25.591 (20.605–51.903)

**Figure 3:**
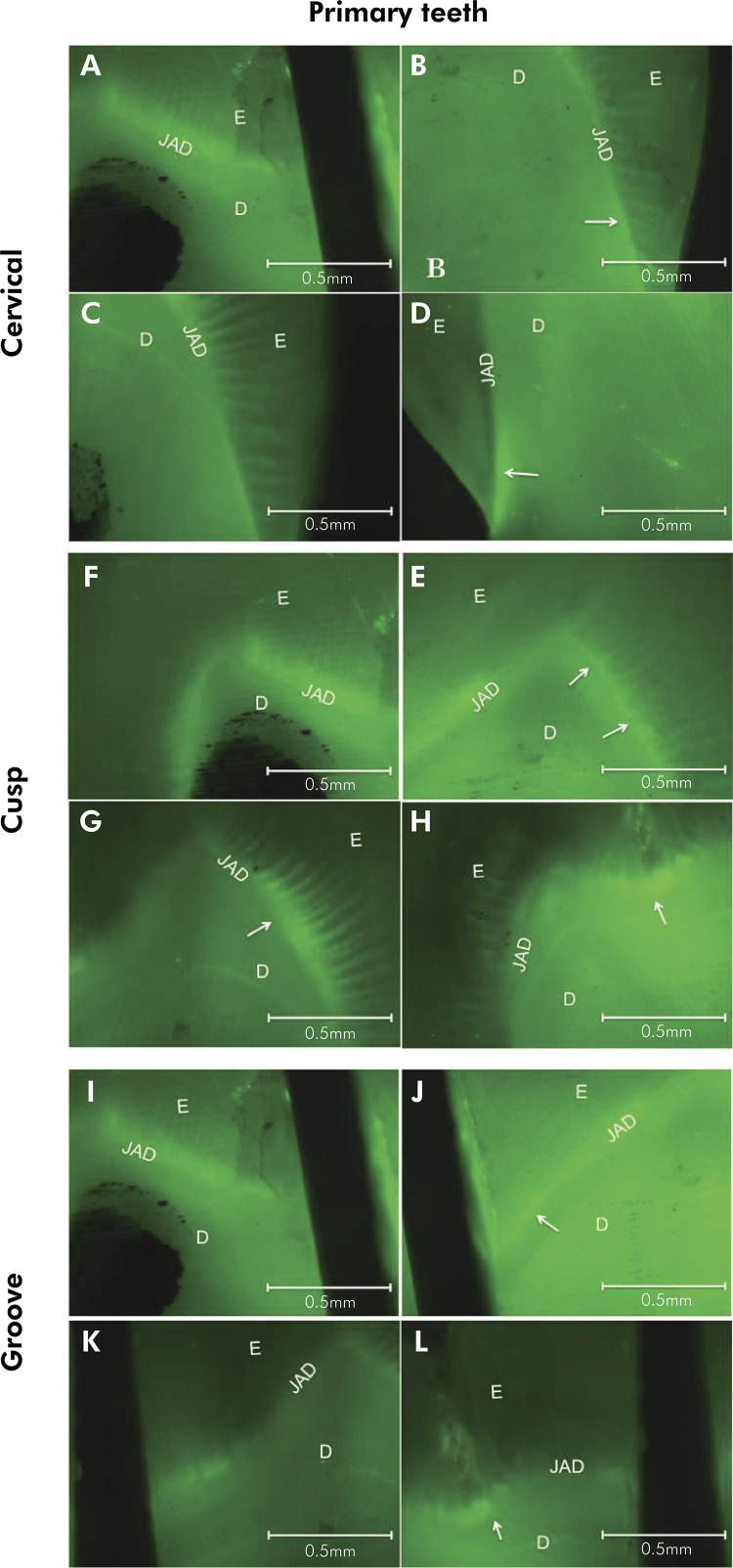
. Fluorescence microscopy revealed gelatinolytic activity in the regions of enamel (E), dentin (D) and dentinoenamel junction (DEJ), in human permanent teeth. (A) Cervical region (5x), showing low activity of MMPs, (B) Cervical region (5x) previously demineralized at a pH of 4.8, showing low activity of MMPs, (C) Cervical region of a sectioned crown irradiated (5x) showing activity of MMPs, (D) Cervical region of an irradiated sectioned crown (5x), previously demineralized at a pH of 4.8 showing activity of MMPs, (E) Cusp (5x) showing low activity of MMPs, (F) Cusp (5x) previously demineralized at a pH of 4.8, showing low MMP activity, (G) Cusp of an irradiated sectioned crown (5x) showing MMPs activity, (H) Cusp of an irradiated sectioned crown (5x) previously demineralized at a pH of 4.8, showing MMP activity, (I) Groove region (5x) showing low MMP activity, (J) Groove region (5x), previously demineralized at a pH of 4.8, showing low activity of MMPs, (K) Groove region of an irradiated sectioned crown (5x), showing activity of MMPs (L) Groove region of an irradiated sectioned crown (5x), previously demineralized at a pH of 4.8, showing activity of MMPs,. Bar = 0.5mm (A, B, C, D, E, F, G, H, I, J, K and L).

In the irradiated permanent teeth, the acid challenge increased the activity of MMPs measured by integrated density of fluorescent signal/mm^2^, showing a statistically significant difference when the teeth were compared with the non-irradiated Group (p < 0.05), in the three regions studied, cervical, cusp and groove. Among irradiated teeth, in those submitted to the acid challenge, the integrated density of fluorescent signal was higher in the cervical, cusp and groove regions (p < 0.05). In non-irradiated permanent teeth, the activity of MMPs was not influenced by demineralization in any of the three areas (Figure 3 and Table 2).

**Table 2:** Comparison of gelatinolytic activity of MMPs in irradiated and / or demineralized permanent teeth, performed by means of in situ zymography. The integrated density of fluorescent signal / mm2 (median and interquartile range) in the cervical region, at the cusp and at the groove was measured at the same depth and in the same area.

Variable	Cervical	Cusp	Groove

	arbitrary units (mm^2^)	arbitrary units (mm^2^)	arbitrary units (mm^2^)
Healthy	15.265 (12.594–17.170)^A^	13.921 (13.056–15.703)^A^	13.758 (13.234–16.315)^A^
Demineralized	21.935 (20.992–26.714)^A.B^	22.784 (21.562–23.482)^A.B^	21.219 (20.407–21.815)^A.B^
Irradiated	29.087 (28.446–29.591)^B^	27.472 (25.870–29.013)^B^	31.707 (28.654–35.317)^B^
Irradiated + Demineralized	40.490 (39.426–42.080)^C^	46.328 (43.097–50.409)^C^	48.285 (46.511–51.093)^C^

Different letters indicate statistically significant differences within the column.

## Discussion

In the present study, the null hypothesis was rejected since activation of MMPs was observed in irradiated permanent teeth when submitted to acid challenge. For primary teeth, the null hypothesis was accepted, as there was no activation of MMPs in teeth irradiated and submitted to acid challenge.

Patients undergoing radiotherapy for the treatment of cancer in the head and neck region may have acute side effects.^13,20,21^ Furthermore, they may have direct effects on mineralized dental tissue due to the increase in the amount of electrons per unit volume in relation to soft tissue, higher dose deposition in the region between tooth and soft tissue, and damage to the salivary glands.^5^ After radiotherapy treatment in teeth, structural, chemical and surface microhardness alterations of the enamel have been observed, in addition to acid solubility. In enamel and dentin, a decrease in proteins was observed, leading to an increase in tissue stiffness close to the DEJ, in addition to tissue disorganization, characterizing an amorphous tooth surface.^5,12, 15,16,22^ In dentin, there was a decrease in biomechanical properties, collagen degradation and presence of broken and/or collapsed dentinal tubules and cracks.^17^

It has been hypothesized that enamel delamination caused by radiation occurs due to lytic changes in collagen polypeptides and activation of MMPs, resulting in the degradation of organic components of enamel and the DEJ.^3-7^ At low pH, demineralization of dentin occurs and collagen fibrils are exposed, and, concomitantly, dentin and/or salivary MMPs are activated.^9^ In this study, acid challenge to permanent teeth was observed to exacerbate the activation of MMPs in the DEJ of irradiated teeth. These data suggested that in addition to being exposed to radiation, the tooth that was subjected to an acid challenge – as usually occurs in post–radiotherapy head and neck patients, who have an increased intake of foods rich in carbohydrates, changes in the oral microbiota, decreased salivary flow and poor oral hygiene – the consequences for dental structures can be even more serious. This emphasizes the importance of a global approach to preventing caries and dental erosion in patients undergoing radiotherapy treatment. In primary teeth, the enzymatic activity was not influenced by irradiation.

A distinct expression of MMPs in primary and permanent teeth, which may explain the difference in enzymatic activity after radiation, has previously been demonstrated.^6,7^ Lower expression of MMP–2 and –20 in primary teeth led to lower gelatinolytic activity in primary teeth, since practically only MMP–9 was present in these teeth.^6^ Whereas in permanent teeth, there were MMPs–2, –9 and –20, which could be activated when exposed to radiation.^24,25^ Therefore, we suggest that since the enzymatic activity of MMPs in these regions was higher in permanent teeth than in primary teeth, permanent teeth may be more vulnerable to enamel delamination and radiation–related caries.

In our study, the acid challenge to which permanent teeth were submitted, increased the activity of MMPs in the cervical and cusp regions of the irradiated teeth. This finding was in agreement with the pathogenesis of radiation–related caries,^6,7^and with the changes that occur in cervical enamel due to the reduced thickness in this region.^25^ This may mean that in a tooth that has undergone first enamel delamination, the presence of an acidic oral environment can contribute to the increase in MMP activity and the appearance of caries lesions characterized by rapid progression and involvement of regions normally considered to be at low risk.

One limitation of this study was that the demineralization protocol used did not simulate the entire process of dental caries or tooth erosion. The strategy used here was to expose teeth that underwent radiotherapy to acid to investigate whether enzymatic activity could be exacerbated. In fact, it was demonstrated that this occurred in permanent but not in primary teeth. Further investigations should be conducted with the use of protocols that simulate erosive and dental caries challenges, to gain better understanding of how activation of enzymes by radiotherapy would impact these processes.

## Conclusion

An acid challenge exacerbated activation of MMPs in DEJ of permanent teeth submitted to irradiation. Whereas in primary teeth, demineralization either combined with irradiation, or not, had no impact on the activity of MMPs.
